# The Genomic Landscape of Antigenic Targets for T Cell-Based Leukemia Immunotherapy

**DOI:** 10.3389/fimmu.2019.02934

**Published:** 2019-12-20

**Authors:** Marie-Pierre Hardy, Krystel Vincent, Claude Perreault

**Affiliations:** Department of Immunobiology, Institute for Research in Immunology and Cancer, Montreal, QC, Canada

**Keywords:** genomics, major histocompatibility complex, mass spectrometry, minor histocompatibility antigen, peptide, proteogenomics, RNA sequencing, tumor-specific antigen

## Abstract

Intensive fundamental and clinical research in cancer immunotherapy has led to the emergence and evolution of two parallel universes with surprisingly little interactions: the realm of hematologic malignancies and that of solid tumors. Treatment of hematologic cancers using allogeneic hematopoietic cell transplantation (AHCT) serendipitously led to the discovery that T cells specific for minor histocompatibility antigens (MiHAs) could cure hematopoietic cancers. Besides, studies based on treatment of solid tumor with *ex vivo*-expanded tumor infiltrating lymphocytes or immune checkpoint therapy demonstrated that anti-tumor responses could be achieved by targeting tumor-specific antigens (TSAs). It is our contention that much insight can be gained by sharing the tremendous amount of data generated in the two-abovementioned universes. Our perspective article has two specific goals. First, to discuss the value of methods currently used for MiHA and TSA discovery and to explain the key role of mass spectrometry analyses in this process. Second, to demonstrate the importance of broadening the scope of TSA discovery efforts beyond classic annotated protein-coding genomic sequences.

## Introduction—Classification of Antigenic Targets

MHC-associated peptides (MAPs) are by-products of protein degradation by proteasomes and other proteases (1). However, while all proteins ultimately undergo proteolytic degradation, only some of them generate MAPs (2, 3). Indeed, the biogenesis of MAPs is regulated by several mechanisms operating at the transcriptional, translational, and post-translational levels (4, 5). Notably, MAPs preferentially derive from proteins degraded during or in the minutes following translation, perhaps by specialized “immunoribosomes” (6).

Four groups of MAPs can be targeted for T-cell based immunotherapy of hematologic cancers: MiHAs, tumor-associated antigens (TAAs), mutated TSAs (mTSAs), and aberrantly expressed TSAs (aeTSAs). MiHAs are encoded by genomic regions with two cardinal features: they contain germline polymorphisms, and they are expressed in both normal and neoplastic cells (7, 8). TAAs derive from unmutated genes that are expressed in normal cells but are overexpressed in cancer cells. In several studies, TAAs have been defined according to the overexpression of the corresponding RNA or source protein. This criterion is not entirely satisfactory considering that (i) T cells see MAPs, not RNA or proteins, and (ii) there is no linear correlation between the abundance of MAPs and the abundance of their source RNA or protein (9–11). Ideally, TAAs should therefore be defined according to MAP abundance on normal vs. neoplastic cells. TSAs are MAPs present only on cancer cells. Identification of mTSAs is relatively straightforward: these MAPs are coded by transcripts bearing somatic mutations such as single nucleotide variants, fusion transcripts, etc. (12, 13). Identification of aeTSAs is more challenging since they are unmutated MAPs that can arise from any genomic region *via* cancer-specific aberrations in gene expression (e.g., alterations in histone or DNA methylation) or splicing (14–17).

Identification of aeTSAs rests on the demonstration that these unmutated MAPs are present only on cancer cells. Two strategies have been used to achieve this goal. The first one hinges on comparison of the immunopeptidome (MAP repertoire) of cancer cells *vs*. that of normal cells (18–20). MAPs found only on cancer cells following mass spectrometry (MS) analyses are labeled as cancer-specific. The limitation of this approach is that some putative aeTSAs may not be entirely cancer-specific because it is currently impossible to obtain the entire MAP repertoire of all types of normal cells. This is particularly true for medullary thymic epithelial cells (mTECs) which have a unique ability to promiscuously express more genes than other types of somatic cells (21). For example, mTECs express several TAAs, that would otherwise qualify as aeTSAs, such as MAGE-A1, MAGE-A3, MAGE-A4, NY-ESO, and CEA (22). Since mTECs induce central immune tolerance, MAPs expressed in mTECs are expected to be poorly immunogenic. It has heretofore been impossible to analyze the immunopeptidome of mTECs because the number of mTECs that can be obtained from a human subject [≈10^6^ cells (23)] is inferior to the number required for comprehensive MS analyses (≈10^8^ cells) and mTECs cannot be expanded *ex vivo*. The second strategy is based on the simple principle that a MAP cannot be present if its source RNA is not expressed. Accordingly, MAPs identified in cancer cells by MS analyses are labeled as aeTSAs only when their source RNA is not expressed in any tissue or organ, including mTECs (14, 16). A caveat of this approach is that presence of a MAP-coding RNA is necessary but not sufficient for expression of this MAP at the peptide level. Hence, this strategy may be too stringent and discard some *bona fide* aeTSAs that would be cancer-specific at the peptide but not the RNA level.

## Identification of Tumor-Specific Antigens

Since the focus of this series is on genetic variants, we will concentrate on TSAs and MiHAs for the rest of this article. This does not mean that TAAs are not interesting targets. The main caveat of TAAs is that they are expected to be poorly immunogenic because they are seen as self-MAPs by T cells. However, transfection of CD8 T cells with a high-affinity WT1-specific TCR yielded promising results in a seminal trial on prevention of AML relapse after allogeneic hematopoietic cell transplantation (24). Notably, no off-target toxicity was observed despite the fact that WT1 is expressed by hematopoietic stem cells, urogenital epithelia, and by mesothelial and fibroblastic cells of the peritoneum, the pleural cavity, and the pericardial cavity (24, 25). Moreover, a vaccine targeting the PR1 TAA also induced PR1-specific immune response in patients with myeloid malignancies (26). Nonetheless, the majority of clinical trials involving TAAs have shown a limited therapeutic potential (27, 28). In contrast to TAAs, TSAs, and MiHAs represent non-self MAPs for autologous and allogeneic T cells, respectively (16, 29, 30). We will limit our review to TSAs and MiHAs presented by MHC class I molecules because the number of studies on MHC II MAPs is relatively limited.

Many studies have been performed in search of TSAs in various tumor types. In most cases, putative TSAs (aka neoantigens) have been identified based on exome sequencing and algorithms that predict MHC binding, without MS validation. This approach is fraught with two major caveats: limited scope and low accuracy.

### Limited Scope

Exons represent only 2% of the genome, whereas 75% of the genome can be transcribed and potentially translated (31). Indeed, MS analyses identified MAPs derived from all sorts of allegedly non-protein-coding regions: introns, 5′UTRs, 3′UTRs, long non-coding RNAs, and intergenic regions (14). Accordingly, many allegedly non-coding regions are in fact protein coding, and translation of “non-coding regions” has been shown to generate numerous MAPs (32–34) some of which were retrospectively identified as targets of TILs and autoreactive T cells (35, 36). In addition, the vast majority of TSAs, and of aeTSAs in particular, derive from allegedly non-coding regions (14). We estimate that mTSAs encoded by canonical exonic open reading frames represent <10% of human TSAs (14). Furthermore, the number of exonic mTSAs should be exceedingly low in leukemias because their mutational load is orders of magnitude lower than that of solid tumors such as melanoma. In fact, to the best of our knowledge, only one mTSA has been unambiguously validated by MS in acute leukemias: this HLA-A^*^02:01–binding peptide results from mutations in the *NPM1* gene that cause the translation of a C-terminal alternative reading frame (15). Another mTSA derived from a BCR-ABL fusion protein was identified *via* MS analyses in 2001 (37), but was not found in a larger cohort of subjects in 2019 (38), and its immunogenicity was called into question (39). The status of this putative TSA therefore remains unclear.

### Low Accuracy

The story of the TEL-AML1 fusion peptide provided one of the first hints that, in the absence of MS validation, predictions based on reverse immunology could be misleading. The TEL-AML1 fusion protein results from a 12; 21 chromosomal translocation and is an important transforming factor in B-cell precursor acute lymphoblastic leukemia. Based on MHC-binding predictions, a TEL-AML1 fusion peptide that could bind to HLA-A^*^02:01 was identified (40). Priming of T cells against this peptide generated cytotoxic T cells that recognized autologous leukemic cells (40). However, when tested experimentally, binding of this peptide to HLA-A^*^02:01 was very weak and its immunogenicity very low. Furthermore, the peptide was not endogenously processed by cells because it was cleaved by proteasomes (41). Hence, the TEL-AML1 fusion peptide was a false discovery, and killing of leukemic cells by T cells primed against the TEL-AML1 fusion peptide (40) was most likely due to the inherent cross-reactivity of T cells which is further amplified in T-cell lines (42). Indeed, positive selection in the thymus preferentially rescues cross-reactive T cells (43) and a single T-cell receptor may recognize more than a million different MAPs (44). Recently, a particularly eloquent demonstration of the low accuracy of mTSA predictions was provided by Löffler at al. who performed comprehensive multi-omic analyses of 16 primary human hepatocellular carcinomas (20). Based on exome and transcriptome sequencing data, MHC-binding algorithms predicted that individual tumors would present an average of 118 exonic mTSAs. Remarkably, none of the 1,888 predicted exonic mTSAs were detected by MS analyses (20). In view of this, the exciting claim that exonic mTSAs can be found in myeloproliferative neoplasms and childhood acute lymphoblastic leukemia must be met with enthusiasm and skepticism since no MS validation was performed on the predicted TSAs (45, 46).

How should we design TSA discovery projects in hematopoietic cancers? We propose that two elements should be taken into consideration. First, we believe that searches limited to exonic TSAs considerably underestimate the diversity of the TSA repertoire (47). According to initial analyses of primary acute lymphoblastic leukemia samples, the vast majority of TSAs are aeTSAs derived from unmutated allegedly non-coding sequences. This analysis led to the discovery that endogenous retroelements (EREs), which are part of our non-coding genome, are a rich source of TSAs. EREs can be defined as remnants of the ancient exogenous retroviruses that infected germ line cells and represent around 43% of the human genome (48). Under physiological conditions, most ERE sequences are silenced, but can be re-expressed in cancer through epigenetic dysregulation of the cancer genome (49). The expression of such sequences can lead to MHC-I presentation of “viral-like” peptides and activate T cells (50). Accordingly, our team identified three ERE-derived TSAs in human ALL samples (14). Moreover, it was shown that the env gene of HERV-K was highly upregulated in AML (51), suggesting that this gene could contribute to AML TSA landscape. Notably, since they are unmutated, aeTSAs can be shared by many patients (52, 53). Second, we strongly suggest that MS analyses should be performed either at the discovery or at the validation stage for all TSAs that might be used as therapeutic targets. Indeed, most bioinformatically “predicted TSAs” not validated by MS analyses probably represent false discoveries. This being said, MS has its own limitations (54). Actually, in the discovery mode, “shotgun MS” is biased toward the most abundant peptides and misses low abundance MAPs (55). Alternatively, targeted MS analyses decreases the detection threshold by about 10-fold, but can be performed only on a limited number of peptides of known amino acid sequence (56). Given the rapid pace of improvements in MS technology it may soon be possible to combine the breadth of shotgun MS with the sensitivity of targeted MS (11, 54).

Once TSAs are discovered, the major remaining challenge is to evaluate their immunogenicity. A recent report suggests that about 80% of virus-derived MAPs validated by MS are immunogenic in mice (57). However, we have no evidence that the rules governing immunogenicity of viral MAPs in mice will apply to TSAs in humans. We reported that the strength of anti-TSA immune response in mice was regulated by two parameters: TSA expression level and the frequency of TSA-responsive T cells in the preimmune (naïve) repertoire (14). However, since only five TSAs were studied, these data should be considered preliminary. For the time being, TSA immunogenicity cannot be predicted, and has to be tested experimentally.

## Identification of Actionable Minor Histocompatibility Antigens

MiHAs are MAPs derived from polymorphic genomic regions. Since over 660 million single nucleotide variants (SNV) and indels have been identified in human populations (58), the potential human MiHA landscape is very broad. Even though MiHA can originate from non-synonymous SNVs in exons or in non-coding regions (32, 59, 60), we will focus herein on exonic MiHAs because they are easier to identify than those generated from atypical transcripts, and probably sufficient to enable immunotherapy of hematologic cancers. Discovery of the first MiHAs in mice (61–64) and humans (65–67) has been a major endeavor, if not a technical tour de force. However, the pace of MiHA discovery increased rapidly with progress in next generation sequencing and MS. For instance, proteogenomic studies led to the identification of over 6,000 MiHAs presented by the most common HLA haplotype in European Americans: HLA-A^*^02:01;B^*^44:03 (60). As for TSAs, MS analyses are instrumental in MiHA discovery/validation because only a small proportion of SNV generate MiHAs (59). Over 90% of MiHA loci are bi-allelic with a dominant allele (that generate MAPs) and a recessive allele (that generates no MAPs) (59, 60, 67). In a few cases, both MiHA alleles are co-dominant. Thus, if we consider MiHAs coded by dominant alleles as winners, it follows that in most cases a single SNV is sufficient to transform winners into losers (the recessive alleles). This is an eloquent reminder that we cannot predict the molecular composition of the immunopeptidome based on our limited understanding of the complexity of the MAP processing pathway (2, 59). More importantly, out of the thousands of MiHAs that we identified, only a minority represent attractive targets for immunotherapy of hematologic tumors with allogeneic T cells (60). Indeed, most MiHAs as non-actionable targets because of their low population frequency and/or their expression in normal epithelial cells.

### Allelic Frequency

As long as it is expressed in tumor cells, a TSA may be considered a potential target. For MiHAs, things are more complicated: in order to be actionable, an MiHA must be present in the recipient and absent in the donor. We refer to this situation as a therapeutic mismatch. The probability to have a therapeutic mismatch is maximal when the allelic frequency of the target MiHA is 0.5 and decreases as the allele frequency approaches the two extremes of 0 and 1 (68). However, because of human population history, most bi-allelic loci have a very common and a very rare allele, with population frequencies of >0.99 and <0.01, respectively (58). MiHAs having an allele frequency of 0.01 or 0.99 would yield a low frequency of therapeutic mismatch: in the first case, MiHA-positive recipients would be rare, whereas in the second case, MiHA-negative donors would be difficult to find. If we consider that actionable MiHA loci must have a minor allele frequency of ≥0.05, then about 92.6% of MiHAs have to be discarded (60).

### Tissue Expression Profile

CD8 T cells targeted to a single MiHA can eradicate tumor cells without causing GVHD, even if expression of the target MiHA is not restricted to hematopoietic cells (69–71). Two elements provide a plausible explanation for the fact that hematopoietic cells are inherently more sensitive than epithelial cells to anti-MiHA T cells: (i) MHC molecules (and therefore MiHAs) are more abundant on hematopoietic cells than epithelial cells and (ii) in one experimental model, MiHA-specific T cells preferentially infiltrated tissues containing VCAM-1^+^ microvessels, that is, the bone marrow and tumor sites (30, 70). Notably, eradication of leukemia cells cannot be achieved by targeting any MiHA. Only MiHAs recognized by CD8 T cells with high functional avidity are effective in mouse models (30, 71–74). As a corollary, we speculate that in clinical trials it may be preferable to target multiple MiHAs simultaneously. Since increasing the number of targeted MiHAs enhances the risk of GVHD (75), it would appear justified to target mainly hematopoietic MiHAs. One additional advantage of targeting non-ubiquitous MiHAs is that “antigen excess” (ubiquitous MiHAs) favor exhaustion of anti-MiHA T cells (76). As for TSAs, the question of MiHA expression by normal cells is not a trivial issue. In practice, we assessed the expression profile of MiHA-coding RNAs in normal tissues, then discarded MiHAs coded by ubiquitously expressed transcripts, and kept only MiHAs preferentially expressed in hematopoietic cells relative to epithelial cells (60). This led to the elimination of two-thirds of MiHAs. In fine, out of the 6,773 MiHAs presented by HLA-A^*^02:01 and HLA-B^*^44:03, only 39 had a minor allele frequency of ≥0.05 and an adequate tissue expression profile (60). This number was sufficient to yield at least one therapeutic mismatch in 90% of related and 98% of unrelated HLA^*^02:01/HLA-B^*^44:03-positive donor-recipient pairs (60). We conclude that the landscape of human exonic polymorphisms is vast enough for MiHA-targeted immunotherapy of practically all subjects suffering from hematologic cancers. In practice, this would require systems-level analyses of the MiHA repertoire presented by other common HLA allotypes.

## Tumor-Specific Antigens and Minor Histocompatibility Antigens—Translational Challenges

In addition to antigen discovery *per se*, scientists involved in the development of TSA- and MiHA-targeted immunotherapies have to address two main challenges: the complexity of precision medicine and the engineering of cost-effective delivery technologies. In the case of TSAs, vaccines appear to be a reasonable delivery strategy to begin with, but the level of precision needed is not inherently obvious. On one side, advocates of individualized vaccines who focus mainly on exonic mTSAs do believe that *de novo* TSA discovery should be performed for individual patients (77, 78). Others, prefer to target shared TSAs (mainly aeTSAs) and rather foresee the development of pre-assembled multi-epitope vaccines containing a series of TSAs presented by specific HLA allotypes (16, 79). In all cases, it is imperative to improve the immunogenicity of TSA vaccines. Accordingly, several different platforms using enhanced vaccine technologies and improved co-stimulatory agents (adjuvants, superantigens, mature dendritic cells) are currently being tested for multiple tumor types including leukemia and lymphoma (28, 77, 80, 81). In the case of MiHAs, whose complexity is more limited than that of TSAs, delivery is probably the major barrier. Almost all pre-clinical research on MiHA-targeted immunotherapy has involved adoptive transfer of allogeneic T cells. Translating this into clinical practice will only be possible when we can count on reliable methods for *ex vivo* generation of sufficient numbers of fit (not exhausted) MiHA-responsive T cells (82–84). Finally, for both TSAs and MiHAs, the strength of anti-leukemic immunotherapy could be further increased with more sophisticated TCR-based therapy using transfected TCRs or bispecific biologics (24, 39, 85).

## Data Availability Statement

Publicly available datasets were analyzed in this study. These data can be found here: MiHA sequences were deposited in the Immune Epitope Database (http://www.iedb.org/) under submission code 1000670. RNA-Seq and exome data were deposited in the NCBI Bioproject database (http://www.ncbi.nlm.nih.gov/bioproject/) under accession code PRJNA286122.

## Author Contributions

M-PH and KV: analysis and interpretation of data, final revisions of the manuscript. CP: financial support and manuscript writing.

### Conflict of Interest

Université de Montréal holds patents and has filed patent applications on minor histocompatibility antigens and tumor-specific antigens. The authors declare that the research was conducted in the absence of any commercial or financial relationships that could be construed as a potential conflict of interest.
